# Topiramate and Kidney Protection: A Serendipitous Connection?

**DOI:** 10.1016/j.ekir.2025.06.012

**Published:** 2025-06-14

**Authors:** Silvio Borrelli, Antonio Russo

**Affiliations:** 1 Nephrology Unit, Department of Advanced Medical and Surgical Sciences, University of Campania “Luigi Vanvitelli”, Naples, Italy; 2 Interdepartmental Program of Headache Medicine and Primary Painful Syndromes, Department of Advanced Medical and Surgical Sciences, University of Campania “Luigi Vanvitelli,” Naples, Italy

See Clinical Research on Page 2642

In recent years, nephrologists have stumbled upon “happy accidents” that have led to unexpected findings of sodium-glucose cotransporter-2 inhibitors having remarkable positive effects on renal and cardiovascular prognosis, extending beyond their primary purpose of reducing hyperglycemia by increasing urinary glucose excretion.^1^

In the latest issue of Kidney International reports, Halimi *et al.*^2^ published an article exploring the potential benefit of topiramate on kidney damage. The findings of that study stem from an extensive retrospective cohort analysis conducted using the TriNetX network, a global, federated platform designed to analyze real-world data from health care organizations worldwide. The study identified 1,805,580 patients suffering from migraine using the International Classification of Diseases 10th Revision code G43, which encompasses a wide variety of migraine phenotypes, including migraine with aura, migraine without aura, hemiplegic migraine, chronic migraine, persistent migraine aura without cerebral infarction, and even the so-called “childhood migraine syndromes.” Among the study population, 317,936 individuals received topiramate and were matched with a corresponding number of patients not receiving it. This matching was accomplished using a sophisticated propensity score method, which accounted for several demographics (e.g., age, sex, race, ethnicity), clinical factors (e.g., estimated glomerular filtration rate [GFR], albuminuria, blood pressure, obesity, diabetes, history of cardiovascular disease, kidney disease, cancer, and other comorbidities), and drugs in therapy (e.g., renin-angiotensin system inhibitors, sodium-glucose cotransporter-2 inhibitors, glucagon-like peptide-1 receptor agonists, statins, antiaggregants, and analgesics) collected at the index date. The study cohort consisted of young adults with an average age of 42.2 years, predominantly women (85.8%), with 9% having diabetes, a mean GFR of 88 ± 25 ml/min per 1.73 m^2^, and < 1% exhibiting albuminuria. Therefore, cohort patients exhibited typical epidemiological characteristics of migraine, though they were at low risk of progressing to end-stage kidney disease.

The authors found that patients treated with topiramate exhibited a reduced adjusted risk of chronic dialysis or kidney transplantation compared with those not receiving topiramate after a median follow-up of 3.5 years (hazard ratio: 0.85; 95% confidence interval: 0.78–0.93). To further examine the potential benefits of topiramate on renal progression, a subgroup of patients (*n* = 162,814) who had a second GFR measurement within 24 to 36 months after the index date was analyzed. In this subcohort, topiramate users exhibited a significant reduction in delta GFR than nonusers (difference in GFR; −0.3 ml/min per 1.73 m^2^; *P* = 0.035), though not clinically significant. In addition, patients treated with topiramate experienced lower all-cause mortality (hazard ratio: 0.83; 95% confidence interval: 0.80–0.86). Therefore, Halimi *et al.*^2^ indicate that topiramate may have a protective effect on the progression of renal failure.

Although the study involved a large population and accounted for adjustments for several known confounders, the retrospective nature of the study design warrants caution when drawing causal conclusions. The first concern is establishing biological plausibility in determining the causal relationship between exposure to topiramate and kidney protection.

Topiramate is a second-generation antiepileptic drug that reduces the frequency, duration, and severity of migraine attacks, supporting its approval as a preventive treatment for both episodic and chronic migraine. Pharmacologically, topiramate blocks voltage-gated sodium channels, reduces calcium influx through high-voltage-activated calcium channels, and inhibits the excitatory neurotransmitter glutamate by antagonizing α-amino-3-hydroxy-5-methyl-4-isoxazole propionate/kainate receptors. These effects collectively suppress neuronal excitation, a process that plays a central role in the development and propagation of migraines. In parallel, topiramate enhances the activity of γ-aminobutyric acid, the principal inhibitory neurotransmitter in the brain. It facilitates γ-aminobutyric acid–A receptor-mediated chloride ion influx, resulting in neuronal hyperpolarization and a reduction in excitability.^3^ In addition to these classic antiepileptic actions, topiramate appears to exert beneficial effects on oxidative stress. Oxidative stress is increasingly recognized as a contributor to the chronic sensitization of pain pathways and to neurogenic inflammation, both of which are implicated in migraine pathogenesis. Topiramate’s antioxidant properties may help mitigate this inflammatory response and restore neuronal homeostasis.^4^

Moreover, topiramate is known to inhibit various carbonic anhydrase isozymes, which can lead to renal tubular acidosis and an increase in urine alkalinity. This alteration in urine pH, along with hypocitraturia and hypercalciuria, may elevate the risk of developing calcium kidney stones. As a result, caution is typically advised when prescribing topiramate to patients with chronic kidney disease (CKD) or a history of calcium nephrolithiasis.^3^

Then, how does it explain the nephroprotective effect of topiramate?

Keeping in mind the renal effects of sodium-glucose cotransporter-2 inhibitors, Halimi *et al.* suggest that topiramate might act on kidney function, decreasing sodium reabsorption at the level of proximal tubule and attenuating intraglomerular pressure through the restoration of tubulo-glomerular feedback. According to this scheme, topiramate reduces sodium proximal tubular reabsorption, inhibiting bicarbonate reabsorption via decreasing carbonic anhydrase activity, resulting in an increase of sodium delivery in macula densa and subsequent adenosine-mediated vasoconstriction of afferent arteriola. The overall effect is tubulo-glomerular feedback restoration and attenuation in glomerular hyperfiltration, which is the primary mechanism of CKD progression. Despite this intriguing pathogenic hypothesis, some experimental data indicate that the tubulo-glomerular feedback response induced by acetazolamide, an inhibitor of carbonic anhydrase, does not persist over time due to intrarenal compensation.^5^

Only a limited number of experimental studies have explored the effect of topiramate on kidney tissue, reporting a reduction in oxidative stress and inflammatory mediators in kidney damage caused by ischemia-reperfusion injury, as well as a protective effect against kidney injury in apoE-deficient mice, which are commonly used as a model for cholesterol-induced atherosclerosis.^6^ In contrast, recent studies have reported a potential effect of γ-aminobutyric acid, glutamate, and glycine on the regulation of renal microvascular function, suggesting caution in the use of topiramate in patients with CKD.^7^ Overall, the current knowledge derived from experimental studies does not provide a solid basis for explaining the mechanisms underlying potential nephroprotection.

In support of the potential benefit of topiramate on kidney function, an extensive survey reported that subjects with migraine and not receiving antimigraine agents had a higher risk of developing CKD^8^; therefore, a better migraine control achieved by topiramate use might be associated with a protective effect on CKD progression, though the mechanism has not been clarified. Although the confounding related to medication use was controlled by matching, some residual bias may still exist, and potentially, the observed effect might be a consequence of the reduction in the long-term use of nephrotoxic drugs, such as nonsteroidal antiinflammatory drugs.

Notably, the authors do not provide any information regarding the dosage of topiramate or the duration of drug exposure, making it challenging to establish the causal relationship between topiramate and kidney protection. This issue is especially relevant because topiramate is often limited by its cognitive side effects (e.g., memory disturbances, attention deficits, and word-finding difficulties, collectively referred to as “dopamax” effects), paresthesia, fatigue, dizziness, and dysgeusia. Real-world data and clinical trials alike have reported high discontinuation rates associated with topiramate use, often exceeding 30% to 40% in 1 year.^9^

In conclusion, research is often driven by scientific curiosity rather than by a structured approach to drug development. Currently, the potential nephroprotective effect of topiramate is in the early stages of the causality process and requires validation through both experimental and clinical evidence to confirm these unexpected findings. Nonetheless, given the high global prevalence and significant burden of disability caused by migraines, clinicians should stay informed about both the benefits and limitations of topiramate, allowing them to make well-informed treatment decisions.

## Disclosure

SB has received participation fees from AstraZeneca, Vantive, CSL Vifor, and Fresenius Kabi. AR has received participation fees and speaker honoraria from Teva, Ely-Lilly, Organon, AbbVie, Lundbeck, Orion Pharma, and Pfizer; and he serves as Chief Editor of “Headache and Neurogenic Pain” Session of Frontiers in Neurology.
